# Penta­carbonyl-2κ^5^ *C*-chlorido-1κ*Cl*-bis­[1(η^5^)-cyclo­penta­dien­yl](μ-1-oxido­ethyl­ene-1:2κ^2^ *O*:*C*)chromium(0)zirconium(IV)

**DOI:** 10.1107/S1600536808042621

**Published:** 2009-01-08

**Authors:** Catharine Esterhuysen, Lizette Retief, Gert J. Kruger, Stephanie Cronje, Helgard G. Raubenheimer

**Affiliations:** a Department of Chemistry and Polymer Science, University of Stellenbosch, Private Bag X1, Matieland 7602, South Africa; b Department of Chemistry and Biochemistry, University of Johannesburg, P O Box 524, Auckland Park, Johannesburg, 2006, South Africa

## Abstract

The title compound, [CrZr(C_5_H_5_)_2_(C_2_H_3_O)Cl(CO)_5_], consists of two metal centres, with a (penta­carbonyl­chromium)oxymethyl­carbene group coordinating as a monodentate ligand to the zirconocene chloride. π-Delocalization through the Zr—O—C=Cr unit is indicated by a short Zr—O distance [2.041 (3) Å] and a nearly linear Zr—O—C angle [170.5 (3)°]. Mol­ecules are aligned with their mol­ecular planes (through Zr, Cl, carbene and Cr) parallel to the *ab* plane. C—H⋯Cl inter­actions result in zigzag chains of mol­ecules propagating parallel to the *b* axis.

## Related literature

For related literature regarding catalytic data of the title compound, see: Sinn *et al.* (1980 ▶); Luruli *et al.* (2004 ▶, 2006 ▶). For other cases of anionic Fischer-type carbenes being used as monodentate ligands, see: Barluenga & Fañanás (2000 ▶). For comparable structures, see: Esterhuysen, Nel & Cronje (2008 ▶); Esterhuysen, Neveling *et al.* (2008 ▶).
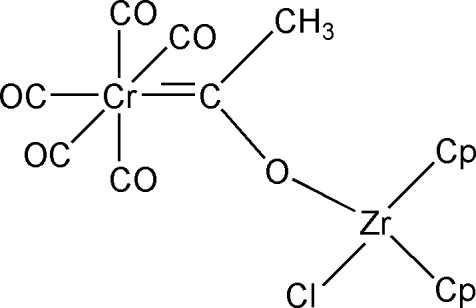


## Experimental

### 

#### Crystal data

[CrZr(C_5_H_5_)_2_(C_2_H_3_O)Cl(CO)_5_]
*M* *_r_* = 491.94
Monoclinic, 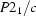

*a* = 12.7395 (7) Å
*b* = 12.1117 (6) Å
*c* = 12.7859 (7) Å
β = 100.826 (5)°
*V* = 1937.71 (18) Å^3^
*Z* = 4
Mo *K*α radiation
μ = 1.27 mm^−1^
*T* = 173 (2) K
0.30 × 0.28 × 0.08 mm

#### Data collection

Philips PW1100 diffractometer
Absorption correction: ψ scan (North *et al.*, 1968 ▶) *T*_min_ = 0.68, *T*_max_ = 0.88
3423 measured reflections
3423 independent reflections
2332 reflections with *I* > 2σ(*I*)
3 standard reflections every 50 reflections intensity decay: none

#### Refinement

*R*[*F* ^2^ > 2σ(*F* ^2^)] = 0.039
*wR*(*F* ^2^) = 0.109
*S* = 1.06
3423 reflections
235 parameters
H-atom parameters constrained
Δρ_max_ = 0.55 e Å^−3^
Δρ_min_ = −0.58 e Å^−3^

### 

Data collection: *PWPC* (Gomm, 1998 ▶); cell refinement: *PWPC*; data reduction: *Xtal3*.4 (Hall *et al.*, 1995 ▶); program(s) used to solve structure: *SHELXS97* (Sheldrick, 2008 ▶); program(s) used to refine structure: *SHELXL97* (Sheldrick, 2008 ▶); molecular graphics: *X-SEED* (Barbour, 2001 ▶; Atwood & Barbour, 2003 ▶); software used to prepare material for publication: *publCIF* (Westrip, 2009 ▶).

## Supplementary Material

Crystal structure: contains datablocks I, global. DOI: 10.1107/S1600536808042621/at2691sup1.cif

Structure factors: contains datablocks I. DOI: 10.1107/S1600536808042621/at2691Isup2.hkl

Additional supplementary materials:  crystallographic information; 3D view; checkCIF report

## Figures and Tables

**Table 1:** Hydrogen-bond geometry (Å, °)

*D*—H⋯*A*	*D*—H	H⋯*A*	*D*⋯*A*	*D*—H⋯*A*
C16—H16⋯Cl1^i^	0.95	2.74	3.581 (8)	149

Symmetry code: (i) 
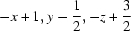
.

**Fig. 1.**
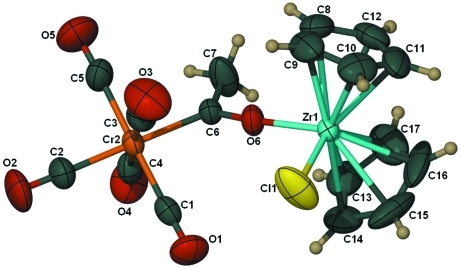
The molecular structure of (I) showing the atomic labelling scheme and displacement ellipsoids drawn at the 50% probability level.

**Fig. 2.**
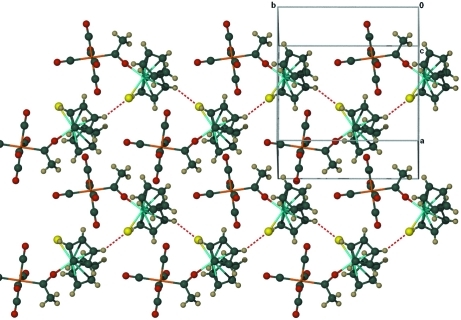
A portion of the packing diagram showing zigzag chains of molecules forming a layer perpendicular to the c axis.

## supplementary crystallographic information

### Comment

Since Cp_2_TiCl_2_ was shown to polymerize ethylene when activated by methylaluminoxane, MAO (Sinn *et al.*, 1980), derivatives of this compound have been synthesized where a Cl ligand was replaced by a monodentate anionic Fischer-type carbene ligand (Barluenga and Fañanás, 2000). We have shown that zirconocene equivalents of this family of homogeneous catalysts, Cp_2_Zr(Cl)OC(*R*)*M*(CO)_5_ (where *M* = W or Cr), catalyze the oligomerization of 1-pentene, as well as the copolymerization of ethene and 1-pentene, in the presence of MAO (Luruli *et al.*, 2004; Luruli *et al.*, 2006). Herein we report the crystal structure of the title zirconocene complex, (I).

In the molecular structure the Zr—O and O—C distances are similar to those found in the equivalent tungsten pentacarbonyl complex (Esterhuysen, Nel & Cronje, 2008). The Zr—O—C angle, on the other hand, is less linear than the previously published tungsten structure [177.4 (7)°], but similar to the hafnocene complex W(CO)_5_C(C_6_H_5_)OHf(C_5_H_5_)_2_Cl (Esterhuysen, Neveling *et al.*, 2008), where the Hf—O—C angle deviates slightly more from linearity [171.4 (3)°]. These results are indicative of π delocalization through the Zr—O—C = W unit.

Molecules are linked by C—H···Cl interactions into zigzag chains along the *b* axis. All molecules in a chain point in the same direction, with their molecular planes parallel. Neighbouring chains in the a-direction have the same orientation, thus forming a layer parallel to the *ab*-plane. Molecules in neighbouring layers in the c-direction have alternating orientations.

### Experimental

A solution of LiCH_3_ (11 ml, 1.5*M* in diethylether, 16.5 mmol) in 10 ml diethylether was added to a well stirred suspension of Cr(CO)_6_ (3.30 g, 15.0 mmol) in 100 ml of diethylether over the period of 1.5 h. The mixture was stripped of solvent *in vacuo*. The residue was dried for 3 h, extracted with cold (273 K), degassed water (1 × 40 ml, 2 × 20 ml) and the formed solution filtered. The aqueous solution was treated with a solution of [NEt_4_]Cl (2.49 g, 15 mmol) in cold, degassed water (4 ml) and the formed precipitate was isolated and dried overnight *in vacuo*. The precipitate was dissolved in warm CH_2_Cl_2_ (5 ml) layered with penatne and cooled to 258 K to yield yellow crystals of (CO)_5_Cr{=C(Me)O}[NEt_4_]. A solution of 0.61 g (2.0 mmol) of the product in 30 ml of CH_2_Cl_2_ was added to a solution of Cp_2_ZrCl_2_ (0.58 g, 2.0 mmol) in 70 ml of diethylether at 233 K over a period of 40 min. AgBF_4_ (0.39 g, 2.0 mmol) was then added to the mixture and stirred for an hour at 233 K. After reaching room temperature the solvent was removed in vacuo and the residue extracted in 5 portions of 10 ml of toluene. The extract was filtered, and the filtrate dried over anhydrous MgSO_4_. The solution was layered with pentane and kept at 258 K to yield orange crystals suitable for X-ray diffraction analysis.

### Refinement

H atoms were positioned geometrically, with C—H = 0.95 Å and 0.98 Å, and constrained to ride on their parent atoms, with *U*_iso_(H) = 1.2 or 1.5*U*_eq_(C). Large anisotropy on atoms C16 and C17 suggests the presence of disorder in the C13–C17 Cp ring, however this could not be modeled. Highest peak: 1.03 Å from Zr1; deepest hole: 1.04 Å from Zr1.

### Crystal data

**Table:**

[CrZr(C_5_H_5_)_2_(C_2_H_3_O)Cl(CO)_5_]	*F*(000) = 976
*M**_r_* = 491.94	*D*_x_ = 1.686 Mg m^−^^3^
Monoclinic, *P*2_1_/*c*	Mo *K*α radiation, λ = 0.71073 Å
Hall symbol: -P 2ybc	Cell parameters from 48 reflections
*a* = 12.7395 (7) Å	θ = 2–17°
*b* = 12.1117 (6) Å	µ = 1.26 mm^−^^1^
*c* = 12.7859 (7) Å	*T* = 173 K
β = 100.826 (5)°	Plate, orange
*V* = 1937.71 (18) Å^3^	0.30 × 0.28 × 0.08 mm
*Z* = 4	

### Data collection

**Table:**

Philips PW1100 diffractometer	2332 reflections with *I* > 2σ(*I*)
Radiation source: fine-focus sealed tube	*R*_int_ = 0.0000
graphite	θ_max_ = 25.0°, θ_min_ = 2.3°
ω–2θ scans	*h* = −15→14
Absorption correction: ψ scan (North *et al.*, 1968)	*k* = 0→14
*T*_min_ = 0.68, *T*_max_ = 0.88	*l* = 0→15
3423 measured reflections	3 standard reflections every 50 reflections
3423 independent reflections	intensity decay: none

### Refinement

**Table:**

Refinement on *F*^2^	Primary atom site location: structure-invariant direct methods
Least-squares matrix: full	Secondary atom site location: difference Fourier map
*R*[*F*^2^ > 2σ(*F*^2^)] = 0.039	Hydrogen site location: inferred from neighbouring sites
*wR*(*F*^2^) = 0.109	H-atom parameters constrained
*S* = 1.06	*w* = 1/[σ^2^(*F*_o_^2^) + (0.053*P*)^2^] where *P* = (*F*_o_^2^ + 2*F*_c_^2^)/3
3423 reflections	(Δ/σ)_max_ < 0.001
235 parameters	Δρ_max_ = 0.55 e Å^−^^3^
0 restraints	Δρ_min_ = −0.58 e Å^−^^3^

### Special details

**Table:**

Geometry. All e.s.d.'s (except the e.s.d. in the dihedral angle between two l.s. planes) are estimated using the full covariance matrix. The cell e.s.d.'s are taken into account individually in the estimation of e.s.d.'s in distances, angles and torsion angles; correlations between e.s.d.'s in cell parameters are only used when they are defined by crystal symmetry. An approximate (isotropic) treatment of cell e.s.d.'s is used for estimating e.s.d.'s involving l.s. planes.
Refinement. Refinement of *F*^2^ against ALL reflections. The weighted *R*-factor *wR* and goodness of fit *S* are based on *F*^2^, conventional *R*-factors *R* are based on *F*, with *F* set to zero for negative *F*^2^. The threshold expression of *F*^2^ > σ(*F*^2^) is used only for calculating *R*-factors(gt) *etc*. and is not relevant to the choice of reflections for refinement. *R*-factors based on *F*^2^ are statistically about twice as large as those based on *F*, and *R*- factors based on ALL data will be even larger.

### Fractional atomic coordinates and isotropic or equivalent isotropic displacement parameters (Å^2^)

**Table:**

	*x*	*y*	*z*	*U*_iso_*/*U*_eq_	
Zr1	0.67690 (3)	0.45977 (3)	0.79870 (3)	0.03838 (16)	
Cr2	0.82371 (6)	0.82936 (6)	0.79664 (6)	0.0441 (2)	
Cl1	0.51592 (14)	0.56328 (16)	0.80604 (18)	0.1054 (7)	
O1	0.6062 (3)	0.7896 (4)	0.6561 (3)	0.0785 (12)	
O2	0.7878 (4)	1.0760 (3)	0.7936 (4)	0.1005 (16)	
O3	0.7167 (4)	0.7914 (4)	0.9852 (4)	0.1065 (17)	
O4	0.9358 (4)	0.8437 (4)	0.6086 (4)	0.0886 (14)	
O5	1.0309 (4)	0.8682 (5)	0.9517 (4)	0.1162 (19)	
O6	0.7769 (3)	0.5916 (3)	0.8006 (3)	0.0532 (9)	
C1	0.6880 (4)	0.8052 (4)	0.7069 (4)	0.0492 (12)	
C2	0.8007 (5)	0.9823 (5)	0.7944 (4)	0.0631 (15)	
C3	0.7572 (5)	0.8072 (5)	0.9152 (5)	0.0627 (15)	
C4	0.8933 (4)	0.8394 (4)	0.6786 (5)	0.0566 (13)	
C5	0.9534 (5)	0.8515 (5)	0.8931 (5)	0.0689 (16)	
C6	0.8496 (4)	0.6625 (4)	0.7962 (4)	0.0449 (11)	
C7	0.9547 (4)	0.6101 (5)	0.7899 (6)	0.086 (2)	
H7A	1.0080	0.6678	0.7871	0.129*	
H7B	0.9469	0.5645	0.7256	0.129*	
H7C	0.9780	0.5639	0.8528	0.129*	
C8	0.8066 (5)	0.4232 (6)	0.9667 (5)	0.0733 (18)	
H8	0.8737	0.4599	0.9772	0.088*	
C9	0.7144 (6)	0.4611 (5)	0.9977 (4)	0.0769 (19)	
H9	0.7065	0.5287	1.0332	0.092*	
C10	0.6349 (5)	0.3814 (6)	0.9672 (4)	0.0758 (18)	
H10	0.5635	0.3850	0.9787	0.091*	
C11	0.6779 (6)	0.2982 (5)	0.9184 (5)	0.0762 (19)	
H11	0.6414	0.2335	0.8893	0.091*	
C12	0.7831 (6)	0.3228 (5)	0.9179 (5)	0.0711 (17)	
H12	0.8315	0.2779	0.8887	0.085*	
C13	0.7269 (6)	0.4442 (6)	0.6179 (5)	0.081 (2)	
H13	0.7843	0.4867	0.6012	0.097*	
C14	0.6228 (6)	0.4758 (6)	0.6006 (5)	0.084 (2)	
H14	0.5943	0.5445	0.5727	0.100*	
C15	0.5653 (7)	0.3857 (10)	0.6326 (6)	0.119 (3)	
H15	0.4901	0.3814	0.6282	0.142*	
C16	0.6386 (11)	0.3060 (7)	0.6711 (6)	0.124 (4)	
H16	0.6228	0.2365	0.6989	0.149*	
C17	0.7361 (9)	0.3420 (7)	0.6632 (5)	0.106 (3)	
H17	0.8011	0.3027	0.6854	0.127*	

### Atomic displacement parameters (Å^2^)

**Table:**

	*U*^11^	*U*^22^	*U*^33^	*U*^12^	*U*^13^	*U*^23^
Zr1	0.0412 (3)	0.0306 (2)	0.0410 (3)	−0.0002 (2)	0.00175 (18)	0.0023 (2)
Cr2	0.0464 (5)	0.0299 (4)	0.0544 (5)	0.0011 (3)	0.0054 (4)	0.0004 (3)
Cl1	0.0717 (11)	0.0871 (13)	0.1666 (19)	0.0361 (10)	0.0464 (12)	0.0408 (12)
O1	0.048 (2)	0.097 (3)	0.084 (3)	0.002 (2)	−0.005 (2)	0.002 (2)
O2	0.121 (4)	0.035 (2)	0.140 (4)	0.013 (2)	0.013 (3)	−0.005 (2)
O3	0.134 (5)	0.122 (4)	0.076 (3)	0.023 (4)	0.053 (3)	0.009 (3)
O4	0.086 (3)	0.096 (3)	0.094 (3)	−0.016 (3)	0.042 (3)	−0.007 (3)
O5	0.077 (3)	0.118 (4)	0.132 (4)	−0.015 (3)	−0.033 (3)	−0.002 (4)
O6	0.052 (2)	0.0333 (18)	0.073 (2)	−0.0062 (16)	0.0079 (17)	−0.0001 (16)
C1	0.058 (3)	0.039 (3)	0.054 (3)	0.008 (2)	0.018 (3)	0.005 (2)
C2	0.074 (4)	0.041 (3)	0.071 (4)	0.004 (3)	0.007 (3)	−0.003 (3)
C3	0.076 (4)	0.055 (3)	0.057 (3)	0.008 (3)	0.010 (3)	−0.005 (3)
C4	0.054 (3)	0.045 (3)	0.071 (4)	−0.008 (3)	0.014 (3)	0.000 (3)
C5	0.061 (4)	0.052 (3)	0.088 (4)	−0.007 (3)	−0.002 (3)	−0.003 (3)
C6	0.044 (3)	0.036 (3)	0.052 (3)	0.000 (2)	0.003 (2)	0.002 (2)
C7	0.048 (3)	0.049 (3)	0.160 (7)	0.008 (3)	0.016 (4)	0.004 (4)
C8	0.073 (4)	0.076 (4)	0.058 (4)	−0.007 (4)	−0.020 (3)	0.016 (3)
C9	0.115 (6)	0.071 (4)	0.041 (3)	0.015 (4)	0.004 (3)	−0.011 (3)
C10	0.083 (5)	0.099 (5)	0.047 (3)	−0.006 (4)	0.019 (3)	0.015 (3)
C11	0.124 (6)	0.049 (3)	0.050 (4)	−0.013 (4)	0.002 (4)	0.014 (3)
C12	0.084 (5)	0.065 (4)	0.061 (4)	0.026 (4)	0.005 (3)	0.020 (3)
C13	0.104 (6)	0.093 (5)	0.048 (3)	−0.021 (4)	0.019 (4)	−0.003 (3)
C14	0.100 (5)	0.094 (5)	0.048 (3)	−0.010 (5)	−0.007 (3)	0.023 (3)
C15	0.104 (6)	0.185 (10)	0.053 (4)	−0.072 (7)	−0.021 (4)	−0.008 (5)
C16	0.232 (12)	0.088 (6)	0.047 (4)	−0.088 (8)	0.009 (6)	−0.022 (4)
C17	0.188 (10)	0.076 (5)	0.058 (4)	0.042 (6)	0.032 (5)	−0.012 (4)

### Geometric parameters (Å, °)

**Table:**

Zr1—O6	2.041 (3)	C6—C7	1.498 (7)
Zr1—Cl1	2.4205 (16)	C7—H7A	0.9800
Zr1—C16	2.463 (7)	C7—H7B	0.9800
Zr1—C17	2.470 (6)	C7—H7C	0.9800
Zr1—C12	2.476 (5)	C8—C12	1.373 (8)
Zr1—C11	2.483 (5)	C8—C9	1.387 (9)
Zr1—C15	2.490 (6)	C8—H8	0.9500
Zr1—C8	2.492 (5)	C9—C10	1.400 (9)
Zr1—C9	2.500 (5)	C9—H9	0.9500
Zr1—C10	2.503 (5)	C10—C11	1.354 (9)
Zr1—C14	2.504 (6)	C10—H10	0.9500
Zr1—C13	2.517 (6)	C11—C12	1.374 (9)
Cr2—C2	1.875 (6)	C11—H11	0.9500
Cr2—C5	1.885 (6)	C12—H12	0.9500
Cr2—C3	1.889 (6)	C13—C14	1.358 (9)
Cr2—C4	1.892 (6)	C13—C17	1.362 (9)
Cr2—C1	1.910 (6)	C13—H13	0.9500
Cr2—C6	2.048 (5)	C14—C15	1.417 (10)
O1—C1	1.135 (6)	C14—H14	0.9500
O2—C2	1.146 (6)	C15—C16	1.368 (13)
O3—C3	1.130 (6)	C15—H15	0.9500
O4—C4	1.131 (6)	C16—C17	1.338 (12)
O5—C5	1.141 (7)	C16—H16	0.9500
O6—C6	1.271 (5)	C17—H17	0.9500
			
O6—Zr1—Cl1	97.24 (10)	C3—Cr2—C6	87.6 (2)
O6—Zr1—C16	130.2 (3)	C4—Cr2—C6	87.8 (2)
Cl1—Zr1—C16	110.7 (3)	C1—Cr2—C6	88.58 (19)
O6—Zr1—C17	100.8 (3)	C6—O6—Zr1	170.5 (3)
Cl1—Zr1—C17	134.4 (2)	O1—C1—Cr2	178.0 (5)
C16—Zr1—C17	31.5 (3)	O2—C2—Cr2	179.2 (6)
O6—Zr1—C12	104.4 (2)	O3—C3—Cr2	178.3 (6)
Cl1—Zr1—C12	133.79 (17)	O4—C4—Cr2	178.7 (5)
C16—Zr1—C12	85.4 (3)	O5—C5—Cr2	177.9 (6)
C17—Zr1—C12	80.9 (2)	O6—C6—C7	112.5 (4)
O6—Zr1—C11	132.73 (19)	O6—C6—Cr2	123.2 (3)
Cl1—Zr1—C11	106.9 (2)	C7—C6—Cr2	124.3 (4)
C16—Zr1—C11	77.9 (3)	C6—C7—H7A	109.5
C17—Zr1—C11	90.6 (3)	C6—C7—H7B	109.5
C12—Zr1—C11	32.2 (2)	H7A—C7—H7B	109.5
O6—Zr1—C15	123.2 (2)	C6—C7—H7C	109.5
Cl1—Zr1—C15	82.4 (3)	H7A—C7—H7C	109.5
C16—Zr1—C15	32.1 (3)	H7B—C7—H7C	109.5
C17—Zr1—C15	52.7 (3)	C12—C8—C9	107.3 (6)
C12—Zr1—C15	116.3 (3)	C12—C8—Zr1	73.3 (3)
C11—Zr1—C15	100.2 (3)	C9—C8—Zr1	74.2 (3)
O6—Zr1—C8	79.46 (18)	C12—C8—H8	126.3
Cl1—Zr1—C8	119.22 (18)	C9—C8—H8	126.3
C16—Zr1—C8	117.0 (3)	Zr1—C8—H8	118.2
C17—Zr1—C8	105.2 (3)	C8—C9—C10	107.4 (6)
C12—Zr1—C8	32.1 (2)	C8—C9—Zr1	73.5 (3)
C11—Zr1—C8	53.3 (2)	C10—C9—Zr1	73.9 (3)
C15—Zr1—C8	148.3 (3)	C8—C9—H9	126.3
O6—Zr1—C9	89.1 (2)	C10—C9—H9	126.3
Cl1—Zr1—C9	87.62 (17)	Zr1—C9—H9	118.3
C16—Zr1—C9	130.9 (3)	C11—C10—C9	107.9 (6)
C17—Zr1—C9	133.9 (2)	C11—C10—Zr1	73.4 (3)
C12—Zr1—C9	53.1 (2)	C9—C10—Zr1	73.6 (3)
C11—Zr1—C9	53.1 (2)	C11—C10—H10	126.0
C15—Zr1—C9	147.1 (3)	C9—C10—H10	126.0
C8—Zr1—C9	32.3 (2)	Zr1—C10—H10	118.8
O6—Zr1—C10	121.38 (19)	C10—C11—C12	108.6 (6)
Cl1—Zr1—C10	81.00 (17)	C10—C11—Zr1	75.1 (3)
C16—Zr1—C10	103.6 (3)	C12—C11—Zr1	73.6 (3)
C17—Zr1—C10	121.9 (3)	C10—C11—H11	125.7
C12—Zr1—C10	52.9 (2)	C12—C11—H11	125.7
C11—Zr1—C10	31.5 (2)	Zr1—C11—H11	117.6
C15—Zr1—C10	114.7 (3)	C8—C12—C11	108.7 (6)
C8—Zr1—C10	53.5 (2)	C8—C12—Zr1	74.6 (3)
C9—Zr1—C10	32.5 (2)	C11—C12—Zr1	74.2 (3)
O6—Zr1—C14	90.23 (19)	C8—C12—H12	125.7
Cl1—Zr1—C14	85.7 (2)	C11—C12—H12	125.7
C16—Zr1—C14	53.6 (3)	Zr1—C12—H12	117.5
C17—Zr1—C14	52.9 (3)	C14—C13—C17	109.2 (7)
C12—Zr1—C14	133.6 (2)	C14—C13—Zr1	73.8 (4)
C11—Zr1—C14	130.8 (2)	C17—C13—Zr1	72.2 (4)
C15—Zr1—C14	33.0 (2)	C14—C13—H13	125.4
C8—Zr1—C14	153.9 (3)	C17—C13—H13	125.4
C9—Zr1—C14	173.1 (2)	Zr1—C13—H13	120.3
C10—Zr1—C14	146.9 (2)	C13—C14—C15	106.2 (7)
O6—Zr1—C13	78.71 (18)	C13—C14—Zr1	74.9 (3)
Cl1—Zr1—C13	115.91 (19)	C15—C14—Zr1	73.0 (3)
C16—Zr1—C13	52.3 (3)	C13—C14—H14	126.9
C17—Zr1—C13	31.7 (2)	C15—C14—H14	126.9
C12—Zr1—C13	108.2 (2)	Zr1—C14—H14	117.5
C11—Zr1—C13	122.3 (2)	C16—C15—C14	107.0 (8)
C15—Zr1—C13	52.6 (3)	C16—C15—Zr1	72.9 (4)
C8—Zr1—C13	122.5 (3)	C14—C15—Zr1	74.1 (4)
C9—Zr1—C13	154.5 (3)	C16—C15—H15	126.5
C10—Zr1—C13	153.4 (2)	C14—C15—H15	126.5
C14—Zr1—C13	31.4 (2)	Zr1—C15—H15	118.6
C2—Cr2—C5	89.1 (2)	C17—C16—C15	108.9 (8)
C2—Cr2—C3	93.4 (2)	C17—C16—Zr1	74.5 (4)
C5—Cr2—C3	88.0 (3)	C15—C16—Zr1	75.0 (5)
C2—Cr2—C4	91.1 (2)	C17—C16—H16	125.5
C5—Cr2—C4	91.6 (3)	C15—C16—H16	125.5
C3—Cr2—C4	175.4 (2)	Zr1—C16—H16	116.8
C2—Cr2—C1	91.2 (2)	C16—C17—C13	108.7 (9)
C5—Cr2—C1	176.2 (2)	C16—C17—Zr1	74.0 (5)
C3—Cr2—C1	88.2 (2)	C13—C17—Zr1	76.1 (4)
C4—Cr2—C1	92.2 (2)	C16—C17—H17	125.7
C2—Cr2—C6	178.9 (2)	C13—C17—H17	125.6
C5—Cr2—C6	91.2 (2)	Zr1—C17—H17	116.3

### Hydrogen-bond geometry (Å, °)

**Table:**

*D*—H···*A*	*D*—H	H···*A*	*D*···*A*	*D*—H···*A*
C16—H16···Cl1^i^	0.95	2.74	3.581 (8)	149

Symmetry codes: (i) −*x*+1, *y*−1/2, −*z*+3/2.
